# Translation and Validation of the Farage Quality of Life (FQoL™) Instrument for Consumer Products into Traditional Chinese

**DOI:** 10.5539/gjhs.v5n1p1

**Published:** 2012-10-16

**Authors:** Miranda A. Farage, Cindy Rodenberg, Jasmine Chen

**Affiliations:** 1 The Procter & Gamble Company, Winton Hill Technical Center, Cincinnati, OH, USA; 2 The Procter & Gamble Company, Beijing, China

**Keywords:** cross-cultural translation, quality of life, emotions, menstrual cycle, menstrual hygiene products, feminine hygiene products, consumer products, FQoL™, well-being, emotion

## Abstract

The Farage Quality of Life™ questionnaire (FQoL™) was developed specifically to assess the impact of consumer products. The objective of this investigation was to achieve a Chinese language instrument. The FQoL™ underwent a forward and backward translation, with cognitive testing by 13 subjects. Slight modifications were made to the instrument, and an implementation study was conducted with 800 participants having a mean (±SD) age of 34.22 (±9.28) years. The subjects were randomly assigned to use 1 of 4 ultra absorbency pad products for the length of one menstrual cycle. Three pads (coded N, S and C) were products currently available on the retail market, a fourth (coded M) was an experimental product improvement on Product N. Subjects were asked to complete the FQoL™ once before (T1) and once after (T2) the start of their period, and the Least Square (LS) Means were determined. Within group comparisons for each item and FQoL™ subscale were conducted by comparing the LS Means for T1 vs. T2. Participants using Product N showed the highest number of significant (p<0.05) changes (11 items), demonstrating these subjects felt worse about items mainly in the subdomains for Emotions, Personal Pleasure, and Physical State. Participants using Product C showed significant changes in 7 items mainly in the subdomains for Emotion and Physical State. Participants using Product S and the experimental Product M showed significant changes in only 4 and 3 individual items, respectively. These were not associated with any particular domain or subdomain. Between group comparisons were conducted by comparing the LS Means for the T2 responses for each group. The group using Product N had LS Mean responses that were significantly worse than the group using Product M for the Emotion, Personal Pleasure and Physical State subdomains, the Energy/Vitality domain, and 2 individual items. The Product S group was worse than the Product M group for 2 individual items. The Product C group was worse than the Product M group for the Personal Pleasure and Physical State subdomains and 5 individual items. We found that the Chinese language FQoL™ detected changes in HRQoL during menstruation compared with before menstruation. Further, the measure was able to detect differences among groups of subjects using different menstrual protection products.

## 1. Introduction

It is well known that chronic illnesses can affect all aspects of daily life, including physical function, mental health, and social function. The current trend is to develop treatment strategies that maximize patient outcomes not only for the specific disease state, but also for all of these broader parameters. As a result, a variety of instruments have been developed recently to monitor patients’ overall function and well-being. Over 700 health-related quality of life (HRQoL) instruments are available across multiple conditions, ranging from cardiovascular disease to skin and eye disorders (PROQOLID, 2012).

Recently, we have extended this approach to evaluate the impact of consumer products (Farage et al., 2010). Consumer products can be broadly defined as products produced for, and purchased by individuals or households for use in their everyday lives, and they include personal care items, such as shampoo and hairspray, and household products, such as detergents and cleaning compounds. The Farage Quality of Life™ questionnaire (FQoL™) was developed specifically to assess the impact of consumer products. The conceptual model is that certain consumer products can have indirect salutary effects on consumers beyond their direct effect on beauty, hygiene, cleanliness, etc. Aspects of HRQoL most likely to be affected include self-image, self-confidence, social function, emotion and well-being.

Initial development of the FQoL™ was described in a recent publication (Farage et al., 2010). This work focused on the impact of the menstrual cycle on HRQoL and the potential influence of a new brand of feminine protection pads. We developed a pool containing 27 items to evaluate overall quality of life, happiness, self-confidence, emotional well-being, physical well-being, and participation in daily routine and recreational activities. In addition, a 3-item Menstrual Module was developed to assess confidence in wearing certain clothes and in going out in public. The initial instrument was subjected to cognitive testing by conducting interviews with 17 women to ensure comprehension and consistent interpretation. Test-retest reliability was evaluated using a group of 20 women who completed the questionnaire twice, 3 days apart. A larger group of 119 women participated in an implementation study in which groups of women used either their usual menstrual pad (control group) or a new brand of menstrual pad (intervention group). These individuals completed the questionnaire 5–7 days before the expected start of their menstrual period, and after the start of menstruation. A factor analysis was conducted to determine the number of latent subscales and, therefore, confirm the domains and subdomains.

The current manuscript describes the continued development of the FQoL™. The primary objective was to determine if the FQoL™ could be adapted for use in non-English-speaking geographies. In addition we wanted to compare overall responses, qualitatively, with those from the English-language version (Farage et al., 2010). The investigation was conducted in China using a similar design to the study conducted in the US. The ultimate goal was to achieve a standard Chinese language instrument that would be semantically and culturally equivalent to the English language instrument. We used the currently accepted approach to developing HRQoL instruments for non-English speaking investigations (summarized by Price et al. (2009)). It involves a forward and backward translation with administration to a small group of subjects followed by cognitive interviews to identify any issues with translation. This approach has been used successfully to develop instruments in a variety of diverse languages, including Chinese (Wu et al., 2012), Portuguese (Freire et al., 2007), Arabic (el Miedany et al., 2003), and Malayalam (Pandey et al., 2004).

## 2. Methods

### 2.1 Translation and Cognitive Testing

#### 2.1.1 Study Design

The questionnaire was translated from English to Chinese, then reverse translated back to English by a different translator in order to confirm that the translation captured the true intent and meaning of each item. After translation, cognitive testing was conducted using a group of 13 women (average age group 26-33 yrs). Participants were recruited by an outside consulting firm (IPSOS China Marketing Research & Consulting Co. Ltd., Guangzh, China). If potential participants were found to meet inclusion/exclusion criteria, they were invited to take part in a 1 hour interview. Inclusion criteria specified that participants have regular menstrual cycles lasting 27-31 days, and have completed high school. Individuals with certain conditions that could significantly impact mood or emotional state were excluded, specifically, individuals being treated for depression, with a history of other mental disorders, or with cancer, diabetes, or HIV. During initial screening, subjects were asked basic demographic information including age, income level and menstrual protection habits. All participants were between the ages of 12 and 50 years, and were required to be women who use regular length menstrual pads as their primary means of feminine protection. Because this was a consumer focus group study, institutional review board (IRB) approval was deemed exempt for this study. Written informed consent was obtained from all subjects.

Participants were asked to take a timed FQoL™, and instructed to circle anything that was unclear to them as they filled out the questionnaire. Responses were given using a five-point Likert scale (“strongly agree”, “mostly agree”, “neither agree nor disagree”, “mostly disagree”, and “strongly disagree”). In addition, most items also included a “does not apply” response option. Interviews were then conducted with the participants to determine their level of comprehension of the questions and whether or not any item was confusing. Interviewers were trained to conduct the cognitive testing in a consistent fashion, and used a script written by the primary investigator during the process. The primary investigator observed all interviews either live or through a recorded video stream. Based on the cognitive study, slight modifications were made to some of the items in the questionnaire.

### 2.2 Implementation Study

#### 2.2.1 Study Design

In each of 4 cities throughout China (Beijing, GuangZhou, LiuZhou, Bengbu), 200 women were recruited for a total of 800 participants (Table 1). In each city, the 200 participants were randomly separated into subgroups of 50 participants each. Each subgroup was assigned to use 1 of 4 ultra absorbency pad products for the length of one menstrual cycle. Thus, 50 women per city in 4 different cities used each product. Three of the test products, i.e., Product code N, Product code S, and Product code C, were currently available on the retail market. These were supplied to the participants as purchased, in the identified package. The fourth product (code M) was a product improvement of product N with a higher absorbency core. This product was supplied in a package identifying it with the product brand name, but not indicating the product contained any improvements.

**Table 1:** Mean age of test group subjects

	Number^a^	Mean age in years (±SD)	Age range (years)
All subjects	800	34.22 (±9.28)	12.01 – 49.25
Test Group 1 - Product N	200	34.43 (±8.63)	13.96 – 49.02
Test Group 2 - Product M	200	33.50 (±9.55)	12.15 – 49.06
Test Group 3 - Product S	200	34.45 (±9.17)	12.37 – 49.25
Test Group 4 - Product C	200	34.48 (±9.77)	12.01 – 49.07

a Each test group contained an equal number of subjects (50) from each of 4 cities.

Subjects were asked to complete the FQoL™ twice: 5–7 days before the expected start of their menstrual period (identified as Time 1, or T1) and 5–7 days after the start of their period (identified as Time 2, or T2). At T1 (when subjects were not menstruating), the recall period was 7 days. At T2 (after the start of menstruation), the recall period was 2 days in order to capture possible changes in the peri-menstrual period. Otherwise, the questionnaires were identical at the 2 time points. As mentioned above, responses were given using a five-point Likert scale, with the addition of a “does not apply” response option for most items.

#### 2.2.2 Statistics

Each response was associated with a numerical value ranging from 1 to 5 with higher scores indicating a worse quality of life. Scores for Item #16 (feeling tired) were reversed for consistency with other item scores. When the “does not apply” option was chosen, the response to that item was eliminated from the data set. For each item, the least squares mean (LS Mean) was determined for T1 and T2, and the change in LS Mean (LS Mean ∆) was calculated (T2 Mean -T1 Mean). In addition to scoring each item individually, items were grouped into the 2 FQoL™ domains and the 7 subdomains. For each grouping, the responses were averaged. If a response was missing for a panelist on any item within a domain or subdomain, or if that panelist chose the “does not apply” option, the missing value was replaced with the average from other items within that domain or subdomain for that panelist. If more than half of the items within a domain or subdomain were missing, responses from that panelist were excluded for that specific domain/subdomain.

We compared within-group changes in responses for each of the 4 product groups. For each of those comparisons, we analyzed the change in response for each item and for each FQoL™ subscale (before vs. during menstruation) using Analysis of Co-Variance (ANCOVA). In addition, we compared the change in response for each pair of products using ANCOVA. All analyses were conducted by using SAS for Windows, version 9.1. We did not adjust our level of significance (p-value) for multiple comparisons.

## 3. Result

### 3.1 Translation and Cognitive Testing

The average completion time of the self-administered questionnaire was approximately 7 ½ minutes. The study results showed that, overall, the questions were understood by the participants. However, there were some translation issues that were identified.

- The neutral response option of “neither agree nor disagree” was found to be awkward when translated to Chinese. For the implementation study this phrase was replaced with a character whose literal translation is “be indifferent”.
- The recall period described as “over the last two days” could not be translated directly since the Chinese character used for “last two days” may also refer to the last few days. For the implementation study the Chinese translation was modified for clarity.
- One of the demographic questions asked for the start of the participants’ last period. The Chinese translation of this phrase was found to be awkward and was modified for clarity.
- Item #6 asked for agreement to the statement, “I have been satisfied with my overall physical appearance”. The phrasing of the translation was thought to be awkward. For the implementation study this item was replaced with, “I have been satisfied with my figure and appearance”.
- Item #17 asked for agreement to the statement, “I have felt calm and peaceful”. The Chinese language does not include the word “calm” as a descriptor for mood. For the implementation study this item was replaced with, “I have felt peaceful”.
- Item #29 asked for agreement to the statement, “I was confident to go out in public”. There is no literal translation for this phrase in Chinese. For the implementation study this item was replaced with, “I was confident to go out to places that have many people”.
- Items #7 and #23 asked for agreement to the statements, “I have been satisfied with the amount of exercise I was able to do”, and “I was able to take part in sports or recreational activities I enjoy”. Some participants expressed confusion about whether these items referred to some physical restriction, or to a general dissatisfaction with their level of physical activity due to time constraints or some other reason. For the implementation study, the decision was made to leave these questions as is.

A number of cultural issues were also identified in the cognitive study.

- One of the demographic questions asked for the participant’s initials as one means of identification. In China, people do not use initials, but instead write out their entire name. For the implementation study participants provided their name, but in the back translation to English it was recorded as a single letter. This, combined with the subject number provided unique identification for the implementation study.
- Dates are given in China as Year/Month/Day, rather than the Western practice of Month/Day/Year. The demographic question on date of birth was changed to be consistent with normal Chinese practice for the implementation study.
- The demographic question of “how many children in the family” was interpreted by some as a question about the number of siblings of the participant. For the implementation study this item was replaced with, “how many children are living with you”.
- The demographic question about marital status had 5 possible responses: married, single, widowed, divorced, or single living with partner. Some women found it offensive to be asked their marital status. In addition, some women who had been divorced or widowed selected the option of “single” in the demographic question about marital status as a reflection of traditional values. This question was not changed for the implementation study.
- Item #11 asked for agreement to the statement, “I have been satisfied with my sex life”. This item was thought to be a matter of privacy, and some participants expressed reluctance to answer such a private question. This item was not changed for the implementation study.

### 3.2 Implementation Study

The response rate for each item is shown in Table 2, along with the response rate for the earlier study conducted in the U.S. With the exception of Item #11-*Sex life*, all questions showed a response rate of >95% in the current Chinese language study. In comparison, there were 5 questions in the earlier English language study where the response rate was <95%. These included Item #11, but also included items in the Routine Activity subdomain (#19-*Go to work or school*) Personal Pleasure subdomain (#21-*Attend public functions*, #23-*Sports/recreation*), and the Menstrual Module (#30-*Confident wearing white*). The item with the lowest response rate in both studies was #11, with 82.00% responding in the China study and 88.14% in the U.S. study.

**Table 2:** Response rates for individual items in the FQoL™

Domain assignments	Subdomain assignments	Item #	Key words ^b^	China study (N=800) % responding	U.S. Study^a^ (N=119) % responding
		1	*Quality of life*	100	100
1	1.1	2	*Happiness*	100	100
1	1.2	3	*Feel good about self*	100	100
2	2.2	4	*Feel physically*	100	100
1	1.2	5	*Self-confidence*	100	100
1	1.2	6	*Physical appearance*	100	100
1	1.2	7	*Amount of exercise*	98.88	97.46
1	1.3	8	*Ability to take care of self*	99.88	100
2	2.3	9	*Personal hygiene*	99.75	100
1	1.2	10	*How clothes fit and look*	99.50	100
2	2.1	11	*Sex life*	82.00	88.14
1	1.1	12	*Manage stress level*	99.75	100
1	1.1	13	*Good mood*	100	100
1	1.1	14	*Positive outlook*	100	100
2	2.2	15	*Energy*	99.75	100
2	2.2	16	*Tired*	97.25	99.15
1	1.1	17	*Calm and peaceful*	99.75	100
2	2.2	18	*Complete household chores*	98.75	100
2	2.3	19	*Go to work or to school*	95.63	83.90
2	2.3	20	*Go where want/need to go*	99.63	100
2	2.1	21	*Attend public functions*	95.50	86.44
2	2.1	22	*Do enjoyable activities*	99.38	100
2	2.1	23	*Sports/recreation*	96.13	93.22
2	2.1	24	*Hobbies*	100	99.15
2	2.1	25	*Enjoy visits with friends/family*	100	98.31
2	2.1	26	*Enjoy time alone*	99.63	100
1	1.3	27	*Feel confident doing things*	99.25	99.15
3		28	*Confident wearing what I want*	99.25	100^c^
3		29	*Confident going out in public*	99.88	100^c^
3		30	*Confident wearing white clothes*	96.63	93.22^c^

The proportion of individuals responding at the baseline (T1) visit with “strongly agree”, “mostly agree”, “neither agree nor disagree”, “mostly disagree”, or “strongly disagree” is provided. Shading represents items with a response rate of <95%.

a (Farage et al., 2010)

b Original English-language key words.

c Results for items 28-30 in the English language study were taken from T2 since these three items were not included on the T1 questionnaire.

We analyzed the change in response for each item and for each FQoL™ subscale from T1 (prior to the start of menstruation) to T2 (during menstruation). Table 3 shows the analysis of these within-group changes in responses for each of the 4 product groups. Participants using Product N showed the highest number of significant changes between T1 and T2. These were mainly in the subdomains for Emotion (#13-*Being in a good mood*, #17-*Feeling calm*), Personal Pleasure (#11-*Sex life*, #21-*Attending public functions*, #23-*Engaging in sports/recreation*, and #24-*Hobbies*), and Physical State (#15-*Having energy* and #18-*Completing household chores*). Significant differences were also seen in single items in the subdomains for Self-image (#3-*Feeling good about oneself*) and Self-competence (#8-*Ability to take care of oneself*). In addition, the responses to both domains (Well-being and Energy/Vitality) and 3 subdomains (Emotion, Personal Pleasure and Physical State) were significantly worse at T2 in this test group.

**Table 3:** Change in response from T1 (prior to the start of menstruation) to T2 (during menstruation)

	Product N				Product M			
	
Domain/Subdomain	LS mean Δ	±	SE	P	LS mean Δ	±	SE	P
1 Well-Being	0.068	±	0.031	***0.027***	-0.009	±	0.031	0.76
1.1 Emotion	0.081	±	0.033	***0.016***	-0.015	±	0.033	0.66
1.2 Self-image	0.053	±	0.035	0.13	-0.015	±	0.035	0.67
1.3 Self-competence	0.067	±	0.043	0.12	0.026	±	0.043	0.55
2 Energy/Vitality	0.080	±	0.030	***0.0073***	-0.003	±	0.030	0.93
2.1 Personal Pleasure	0.100	±	0.035	***0.0046***	-0.004	±	0.035	0.91
2.2 Physical State	0.100	±	0.035	***0.0044***	-0.012	±	0.035	0.73
2.3 Routine activity	0.024	±	0.037	0.52	0.019	±	0.037	0.61
3 Menstrual Module	0.001	±	0.045	0.98	-0.049	±	0.045	0.28

Item								
*1-Quality of life*	0.158	±	0.055	***0.0039***	0.138	±	0.055	***0.011***
*2-Happiness*	0.080	±	0.060	0.18	0.017	±	0.060	0.78
*3-Feel good about self*	0.089	±	0.042	***0.035***	0.044	±	0.042	0.29
*4-Feel physically*	0.088	±	0.054	0.10	-0.052	±	0.054	0.34
*5-Self-confidence*	0.069	±	0.053	0.19	-0.006	±	0.053	0.91
*6-Physical appearance*	0.040	±	0.050	0.42	-0.019	±	0.050	0.70
*7-Amount of exercise*	0.006	±	0.062	0.93	-0.033	±	0.061	0.59
*8-Ability to take care of self*	0.109	±	0.048	***0.024***	0.007	±	0.048	0.88
*9-Personal hygiene*	0.044	±	0.045	0.33	0.134	±	0.045	***0.0033***
*10-How clothes fit and look*	0.058	±	0.050	0.25	-0.046	±	0.050	0.37
*11-Sex life*	0.231	±	0.066	***0.0005***	0.263	±	0.065	**<0.0001**
*12-Manage stress level*	0.035	±	0.044	0.43	-0.020	±	0.044	0.65
*13-Good mood*	0.132	±	0.048	***0.0059***	-0.010	±	0.048	0.83
*14-Positive outlook*	0.058	±	0.046	0.21	-0.046	±	0.046	0.31
*15-Energy*	0.154	±	0.057	***0.0067***	-0.030	±	0.057	0.59
*16-Tired*	0.024	±	0.067	0.73	0.021	±	0.067	0.76
*17-Calm and peaceful*	0.102	±	0.045	***0.023***	0.002	±	0.045	0.97
*18-Complete household chores*	0.125	±	0.050	***0.013***	-0.004	±	0.050	0.94
*19-Go to work or to school*	-0.044	±	0.051	0.39	-0.010	±	0.051	0.84
*20-Go where want/need to go*	0.055	±	0.056	0.32	-0.065	±	0.056	0.25
*21-Attend public functions*	0.124	±	0.063	***0.048***	-0.024	±	0.062	0.71
*22-Do enjoyable activities*	0.021	±	0.052	0.69	-0.078	±	0.052	0.14
*23-Sports/recreation*	0.134	±	0.066	***0.042***	-0.028	±	0.065	0.66
*24-Hobbies*	0.120	±	0.052	***0.021***	0.000	±	0.052	1.00
*25-Enjoy visits with friends/family*	0.017	±	0.049	0.73	-0.055	±	0.049	0.26
*26-Enjoy time alone*	0.042	±	0.059	0.48	-0.076	±	0.059	0.20
*27-Feel confident doing things*	0.035	±	0.055	0.52	0.042	±	0.055	0.44
*28-Confident wearing what I want*	0.033	±	0.058	0.57	0.015	±	0.058	0.80
*29-Confident going out in public*	-0.015	±	0.052	0.78	-0.102	±	0.052	**0.049**
*30-Confident wearing white clothes*	0.006	±	0.065	0.93	-0.064	±	0.065	0.32

	Product S				Product C			
	
Domain/Subdomain	LS mean Δ	±	SE	P	LS mean Δ	±	SE	P
1 Well-Being	0.018	±	0.031	0.57	0.057	±	0.031	0.063
1.1 Emotion	-0.006	±	0.033	0.85	0.062	±	0.033	0.062
1.2 Self-image	0.041	±	0.035	0.24	0.054	±	0.035	0.12
1.3 Self-competence	0.030	±	0.043	0.48	0.042	±	0.043	0.33
2 Energy/Vitality	0.040	±	0.030	0.18	0.076	±	0.030	***0.011***
2.1 Personal Pleasure	0.037	±	0.035	0.30	0.095	±	0.035	***0.0070***
2.2 Physical State	0.056	±	0.035	0.11	0.113	±	0.035	***0.0013***
2.3 Routine activity	0.065	±	0.038	0.09	-0.012	±	0.037	0.74
3 Menstrual Module	-0.009	±	0.045	0.84	0.079	±	0.045	0.08

Item								
*1-Quality of life*	0.054	±	0.055	0.33	0.040	±	0.055	0.46
*2-Happiness*	0.002	±	0.060	0.97	0.026	±	0.060	0.67
*3-Feel good about self*	0.065	±	0.042	0.12	0.041	±	0.042	0.33
*4-Feel physically*	0.000	±	0.054	1.00	0.044	±	0.054	0.41
*5-Self-confidence*	0.047	±	0.053	0.38	0.039	±	0.053	0.46
*6-Physical appearance*	0.048	±	0.050	0.34	0.086	±	0.050	0.09
*7-Amount of exercise*	-0.039	±	0.061	0.52	-0.030	±	0.062	0.63
*8-Ability to take care of self*	0.040	±	0.048	0.41	0.029	±	0.048	0.55
*9-Personal hygiene*	0.180	±	0.045	***<0.0001***	0.068	±	0.045	0.13
*10-How clothes fit and look*	0.103	±	0.051	***0.043***	0.116	±	0.050	***0.021***
*11-Sex life*	0.278	±	0.065	***<0.0001***	0.388	±	0.065	***<0.0001***
*12-Manage stress level*	-0.045	±	0.044	0.30	0.105	±	0.044	***0.016***
*13-Good mood*	0.014	±	0.048	0.77	0.129	±	0.048	***0.0072***
*14-Positive outlook*	0.039	±	0.046	0.40	0.035	±	0.046	0.44
*15-Energy*	0.170	±	0.057	***0.0027***	0.137	±	0.057	***0.015***
*16-Tired*	0.060	±	0.067	0.37	0.162	±	0.067	***0.017***
*17-Calm and peaceful*	-0.021	±	0.045	0.64	-0.002	±	0.045	0.96
*18-Complete household chores*	-0.001	±	0.050	0.99	0.083	±	0.051	0.10
*19-Go to work or to school*	-0.016	±	0.051	0.76	-0.062	±	0.051	0.22
*20-Go where want/need to go*	0.046	±	0.056	0.41	-0.042	±	0.056	0.45
*21-Attend public functions*	0.005	±	0.063	0.94	0.090	±	0.063	0.15
*22-Do enjoyable activities*	-0.029	±	0.053	0.59	0.020	±	0.052	0.70
*23-Sports/recreation*	0.015	±	0.065	0.82	0.063	±	0.065	0.33
*24-Hobbies*	0.049	±	0.052	0.34	0.046	±	0.052	0.37
*25-Enjoy visits with friends/family*	-0.024	±	0.049	0.62	0.063	±	0.049	0.20
*26-Enjoy time alone*	0.014	±	0.059	0.81	0.019	±	0.059	0.74
*27-Feel confident doing things*	0.036	±	0.055	0.51	0.048	±	0.055	0.38
*28-Confident wearing what I want*	0.065	±	0.058	0.26	0.144	±	0.058	***0.013***
*29-Confident going out in public*	-0.015	±	0.052	0.77	0.052	±	0.052	0.31
*30-Confident wearing white clothes*	-0.050	±	0.065	0.44	0.065	±	0.064	0.31

Items were scored on a 1-5 scale, with higher scores indicating worse health-related quality of life (scores for Item 16 were reversed for consistency with other item scores). For each item, the least squares mean (LS Mean) was determined for T1 and T2, and the change in LS Mean (LS Mean ∆) was calculated (T2 Mean -T1 Mean). The p value was determined using ANCOVA. Bold italic font indicates significant (p≤0.05) worsening from baseline. Bold font with grey fill indicates significant (p≤0.05) improvement from baseline.

Participants using the experimental Product M showed the fewest number of significant changes for the worse, and included Items #1-*Quality of life*, #9-*Personal hygiene* and #11-*Sex life* (the latter two in the Energy/Vitality domain). One item in the Menstrual Module showed significant improvement in the score for the Product M group: Item #29-*Confident going out in public*.

Participants using Product S showed significant changes in 4 individual items: three in the Energy/Vitality domain (#9-*Personal hygiene*, #11-*Sex life*, and #15-*Having energy*), and one in the Well-being domain (#10-*How clothes fit and look*). Participants using Product C showed significant changes in items in the subdomains for Emotion (#12-*Managing stress* and #13-*Being in a good mood*) and Physical State (#15-*Having energy* and #16-*Feeling tired*). Single items were also significantly worse in the subdomains for Personal Pleasure (#11-*Sex life*) and Self-image (#6-*Satisfaction with appearance*), and in the Menstrual Module (#28-*Confidence wearing what I want*). The responses to the Energy/Vitality domain and the Personal Pleasure and Physical State subdomains were significantly worse at T2 in this test group.

We compared the response for each pair of products at T2 (after the start of menstruation) in order to determine if there were any product related significant differences. Table 4 shows the analysis of these between group comparisons. The comparisons between groups using products N and M showed that in all cases where the difference was significant those using product M had a lower LS Mean indicating a better quality of life. These included the Energy/Vitality domain, the Emotion, Personal Pleasure and Physical State subdomains, and individual Items #13-*Good mood* and #15-*Energy* within the subdomains for Emotion and Physical State, respectively. Similarly, in all cases where the LS Mean for the Product M group was significantly different from that for the Product S group or the Product C group, the product M group had a lower LS Mean. For the Product M to Product S comparison this included Items #10-*How clothes fit and look* in the Self-image subdomain, and #15-*Energy* in the Physical State subdomain. For the Product M to Product C comparison this included Item #10-*How clothes fit and look* in the Self-Image subdomain, Items #12-*Manage stress level*, #13-*Good mood* in the Emotion subdomain, #15-*Energy* in the Physical State subdomain, and #29-*Confident going out in public* in the Menstrual Module. In addition, the Personal Pleasure and Physical State subdomains were significantly different for the Products M and C comparison. In addition, the T2 LS Mean for Product N was significantly lower than that for Product S for Item #9-*Personal hygiene*, and the T2 LS Mean for Product S was significantly lower than that Product C for Item #12-*Manage stress level*.

**Table 4:** Comparison between test groups of T2 (during menstruation) responses

Keywords			Product M		Product S		Product C	

		T2 LS Mean	Difference	P	Difference	P	Difference	P
**Domain/Subdomain**								
1.1 Emotion	Product N	2.1911	0.0958	***0.043***	0.0874	0.0649	0.0186	0.6942
	Product M	2.0953			-0.0085	0.858	-0.0772	0.1029
	Product S	2.1038					-0.0688	0.1463
	Product C	2.1725						
2 Energy/Vitality	Product N	2.2085	0.0825	***0.0495***	0.0394	0.3477	0.0039	0.9254
	Product M	2.126			-0.0431	0.3051	-0.0786	0.0614
	Product S	2.169					-0.0355	0.398
	Product C	2.2045						
2.1 Personal Pleasure	Product N	2.2601	0.104	***0.0366***	0.0631	0.2058	0.005	0.9194
	Product M	2.1561			-0.0409	0.4105	-0.099	**0.0464**
	Product S	2.1971					-0.058	0.2434
	Product C	2.2551						
2.2 Physical State	Product N	2.4306	0.1126	***0.0235***	0.0442	0.3733	-0.0131	0.7912
	Product M	2.318			-0.0684	0.168	-0.1257	**0.0114**
	Product S	2.3864					-0.0573	0.2483
	Product C	2.4437						
Item								
9 *Personal hygiene*	Product N	1.529	-0.0899	0.1619	-0.1357	**0.035**	-0.0243	0.7049
	Product M	1.619			-0.0457	0.4767	0.0656	0.3072
	Product S	1.6647					0.1114	0.0831
	Product C	1.5533						
10 *How clothes fit and look*	Product N	1.9635	0.1032	0.1482	-0.045	0.5286	-0.0586	0.4107
	Product M	1.8603			-0.1481	**0.0385**	-0.1617	**0.0233**
	Product S	2.0084					-0.0136	0.849
	Product C	2.022						
12 *Manage stress level*	Product N	2.1929	0.0545	0.3777	0.0795	0.1975	-0.0706	0.2528
	Product M	2.1385			0.0251	0.6853	-0.125	**0.0435**
	Product S	2.1134					-0.1501	**0.0153**
	Product C	2.2635						
13 *Good mood*	Product N	2.0033	0.1421	***0.0362***	0.118	0.0816	0.003	0.9652
	Product M	1.8612			-0.0241	0.7221	-0.1392	**0.0404**
	Product S	1.8853					-0.1151	0.0896
	Product C	2.0003						
15 *Energy*	Product N	2.2913	0.1846	***0.0218***	-0.0155	0.8467	0.017	0.8316
	Product M	2.1067			-0.2001	**0.0127**	-0.1676	**0.0367**
	Product S	2.3068					0.0325	0.6841
	Product C	2.2742						
29 *Confident going out in public*	Product N	2.0153	0.0874	0.2331	0.0005	0.9942	-0.0668	0.3618
	Product M	1.9279			-0.0869	0.2365	-0.1542	**0.0355**
	Product S	2.0148					-0.0674	0.3586
	Product C	2.0821						

Items were scored on a 1-5 scale, with higher scores indicating worse health-related quality of life (scores for Item 16 were reversed for consistency with other item scores). For each item, the least squares mean (LS Mean) was determined for T2. The difference in the LS Mean at T2 was calculated by subtracting the mean of the product in the column from the mean of the product in the row (e.g., Product N – Product M, or 2.1911 – 2.0953 = 0.0958). Pairs of product legs were compared using ANCOVA. Bold italic font indicates the result for the product in the row was significantly (p<0.05) worse than that of the column. Bold font with grey fill indicates result of the product in the row was significantly (p<0.05) better than that of the column.

## 4. Discussion

With about 1.3 billion people, China represents approximately 20% of the world’s population. Therefore, developing HRQoL instruments for use in China is key. A number of investigators have reported the translation into Chinese of HRQoL instruments to evaluate patients with various disease states. Zhao and Kanda (2000) reported on a translation of the European Organization for Research and Treatment of Cancer Quality of Life Core Questionnaire (EORTC QLQ-C30, version 2.0). Chinese versions of the Functional Assessment of Cancer Therapy-General (FACT-G) have been reported by Yu et al. (2000), Lau et al. (2002), and Cheung et al. (2003). Lai et al. (2010) described the development of a Chinese version of the Quality of Life Questionnaire of the European Foundation for Osteoporosis (QUALEFFO-31). Huang et al. (2007) reported on the development of a Chinese version of the Irritable Bowel Syndrome–Quality of Life questionnaire (IBS–QOL) instrument. Development and validation of a Chinese version of the dizziness handicap inventory (DHI) was reported by Poon et al. (2004). Yeung et al. (2006) reported on the Gastrointestinal Quality of Life Index (C-GIQLI) for use in patients with gastric tumors after gastrectomy. Recently, Wu et al. (2012) reported on the successful adaptation of life assessment instrument for children with hemophilia.

These are only some examples where authors used the same basic approach to developing the Chinese language instrument, i.e., forward and backward translation, with pilot testing using a small number of subjects followed by interviews to ensure comprehension.

Some issues associated with translating HRQoL instruments from English into Chinese were reviewed by Cheung and Thumboo (2006). These authors identified potential issues in several broad areas. The first area is in translation and semantic equivalence. Often, words or phrases can have multiple meanings in English leading to more than one potential translation of the phrase into Chinese. Such ambiguities are compounded if the individual Chinese translations also have more than one meaning. Potential issues may also arise with regard to conceptual equivalence for individual items. For example, common activities may differ in various geographies. Westerners may play golf as a form of exercise, whereas the Chinese may practice Tai Chi. Items that are culturally specific may not produce a comparable result if translated directly into Chinese. Cultural and attitudinal differences may also exist. Cheung and Thumboo (Cheung and Thumboo, 2006) reported that between 22% and 50% of responders in Asian geographies did not answer the question “I am satisfied with my sex life” in an HRQoL instrument. This is compared to a 7% non-response rate in the US.

In this study, the response rate was better to the question about sex life (Item #11), with 82% responded, as shown in Table 2. In fact, if younger subjects (i.e., 62 subjects of <20 years of age) are removed from the data set, the response rate to the question about sex life was 87.53% for participants ≥20. This is very similar to the response rate of 88.18% that we observed in the earlier, English language study.

The response rate to all other questions in the Chinese language study was >95%. Interestingly, in the US study there were several additional items where the response rate was <95% (Items #19, #21, #23 and #30.)

**Figure 1:**
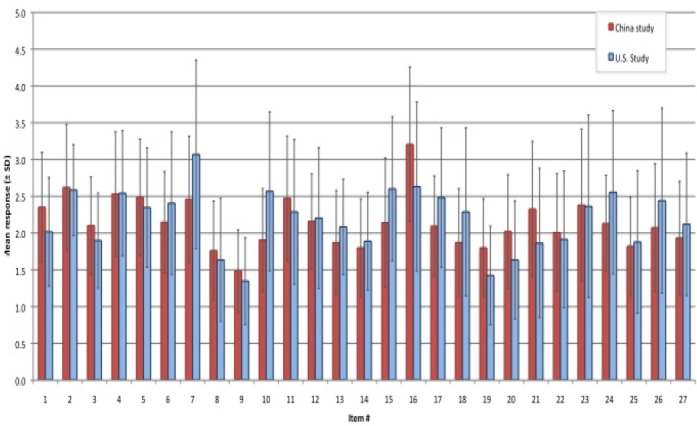
[MAF1]Comparison of mean responses at T1 (before the start of menstruation): Chinese language vs. English language

In the current investigation, the cognitive interviews confirmed that overall comprehension of the individual items was high. Most of the issues identified in the discussions were in regard to awkward translations rather than ambiguous meanings of the translations. Relatively few modifications were required to the individual items in the English language instrument. The semantic equivalence of the translated instrument is supported by a comparison of the overall responses. Figure 1 compares the mean of the baseline (or T1) responses of women from the current study to those of the study conducted in the U.S. using the English language version (Farage et al., 2010). For all but three items (#7, #10 and #16), the difference in the mean response was less than a half point.

For both the current study and the earlier English language study (Farage et al., 2010), the mean response (±SD) for each item in the FQoL™ was calculated for T1 (i.e., the baseline visit prior to the start of menstruation). T1 results for items 28-30 in the English language study were not available since these three items were not included on the baseline questionnaire in this study. Our study had couple of limitations. Due to logistics reasons, it was necessary to supply the commercially- available products to the test subjects as purchased in the identified package. This may have imparted some bias to the study due to the reputation of the various brands. Importantly, Products N and M were supplied in identically labeled packaging. Therefore, comparisons between these two products can be interpreted with greater confidence.

In addition, the initial cognitive test was given during the period of Chinese New Year celebrations (also called the Spring Festival). This is an important traditional Chinese holiday marking the end of the winter season, and a number of participants indicated that their mood was substantially altered during the Spring Festival celebration. It is unlikely that this affected the cognitive understanding of the participants. However, this time of year may lead to abnormal results for items intended to measure mood and social activities. It will be important in future studies in China to avoid this time of year when administering the instrument.

## 5. Conclusion

The goal of this current investigation was to establish an instrument to measure the QoL impact of consumer products in China that is semantically and culturally equivalent to the English language instrument. We found that the Chinese language FQoL™ detected significant changes in HRQoL for the worse during menstruation compared with before menstruation in several individual items and some domains/subdomains. The measure was able to detect differences between groups of subjects using 4 different menstrual protection products: 3 products currently available on the market (Product codes N, S, and C), and one experimental product with a higher absorbancy core (Product code M). The group using the experimental product (M) showed the fewest number of significant changes for the worse compared to the other 3 test groups. In paired comparisons, each of the 3 groups using the currently-available products showed a higher number of signficant changes for the worse compared to the group using the experimental product (M). Qualitatively, the mean baseline responses for the Chinese-language responders were comparable to those of the English-language study previously published (Farage et al., 2010).
